# Ileus from a Turning or Twisting of the Bowel on Its Axis

**Published:** 1844-06-29

**Authors:** 


### ILEUS FROM A TURNING OR TWISTING OF THE BOWEL ON ITS AXIS.

A man, in loading his waggon with clover, had occasion to mount and jump down again repeatedly, after which he was taken with colic pains and obstinate constipation. All remedies and means were vain, and the patient died on the fifteenth day of his illness. The ileum at its lower extremity, where it terminated in the cœcum, was found simply turned or *twisted upon its axis.* The turn was single, but the piece of intestine was as securely confined as if it had had a noose of thread cast over it. The small intestines were slightly reddened, and excessively distended with gas; the great intestine, on the contrary, was pale and contracted. The twisted portion of gut being set free, it was found quite pervious and uninjured. Would the use of quicksilver have been of any service in this case?—*London Med. Gaz. from Casper's Wochenschrift.*

[A similar case of twisting of the intestine upon its axis, in which the ileum was found to have made *three entire revolutions*, is contained in the *Examiner* of the 20th of April last, reported by Dr. McPheeters of St Louis.]

---

Dr. Fleming, an eminent Veterinary Surgeon, and Director of the Veterinary School of Rosenthell, lately died of hydrophobia after twenty-four hours of frightful sufferings. Dr. Fleming had never been bitten by any animal affected with madness, but about three years back he dissected a dog which had died mad.—*London Medical Times.*

